# Robotic traction-assisted en bloc endoscopic submucosal dissection of early esophageal cancer on the left lateral wall

**DOI:** 10.1055/a-2948-3371

**Published:** 2026-09-22

**Authors:** Peng Shen, Rui Xie, HongGang Wang, Yin Xu, XiaoZhong Yang, Yang Jiang, WeiJie Dai

**Affiliations:** 1 Department of Gastroenterology 91596 The Affiliated Huai’an No.1 People’s Hospital of Nanjing Medical UniversityHuai'anJiangsuChina

Endoscopic submucosal dissection (ESD) on the left lateral wall of the lower esophagus is challenging: gravity-dependent fluid pooling obscures the submucosal layer, increasing the risk of bleeding and incomplete resection. EndoFaster (ROBO Med, Shenzhen, China), a flexible single-arm transluminal endoscopic robot, has shown safety and feasibility in the gastric fundus, lower rectum, and descending duodenum. 1​ 2​ ​3​ We present robotic traction-assisted en bloc ESD for an early esophageal cancer at this site, where pooled fluid defeats conventional long-axis traction.

A 75-year-old man underwent gastroscopy, which revealed a 4.0×3.0 cm early esophageal cancer involving nearly half of the circumference, 35–39 cm from the incisors ( Fig. 1a ); biopsy showed squamous high-grade intraepithelial neoplasia. During EndoFaster-assisted ESD, after submucosal injection and hybrid-knife circumferential incision ( Fig. 1b ), the forceps, hood-mounted on the endoscope tip, was set at the 12 o’clock position. Pooled fluid persistently obscured the submucosal layer ( Fig. 1c ). The forceps grasped the mucosal edge and applied sustained upward traction at a right angle to the muscularis propria, exposing the submucosal layer ( Fig. 1d ) and blood vessels ( Fig. 1e ). Dynamic repositioning enabled multi-directional traction, precise hemostasis, and systematic dissection, completed in 12.6 minutes without perforation or muscle injury ( Fig. 1f , Video 1 ). The patient was discharged on postoperative day 3. Pathology confirmed en bloc R0 resection ( Fig. 2 ).

**Fig. 1:**
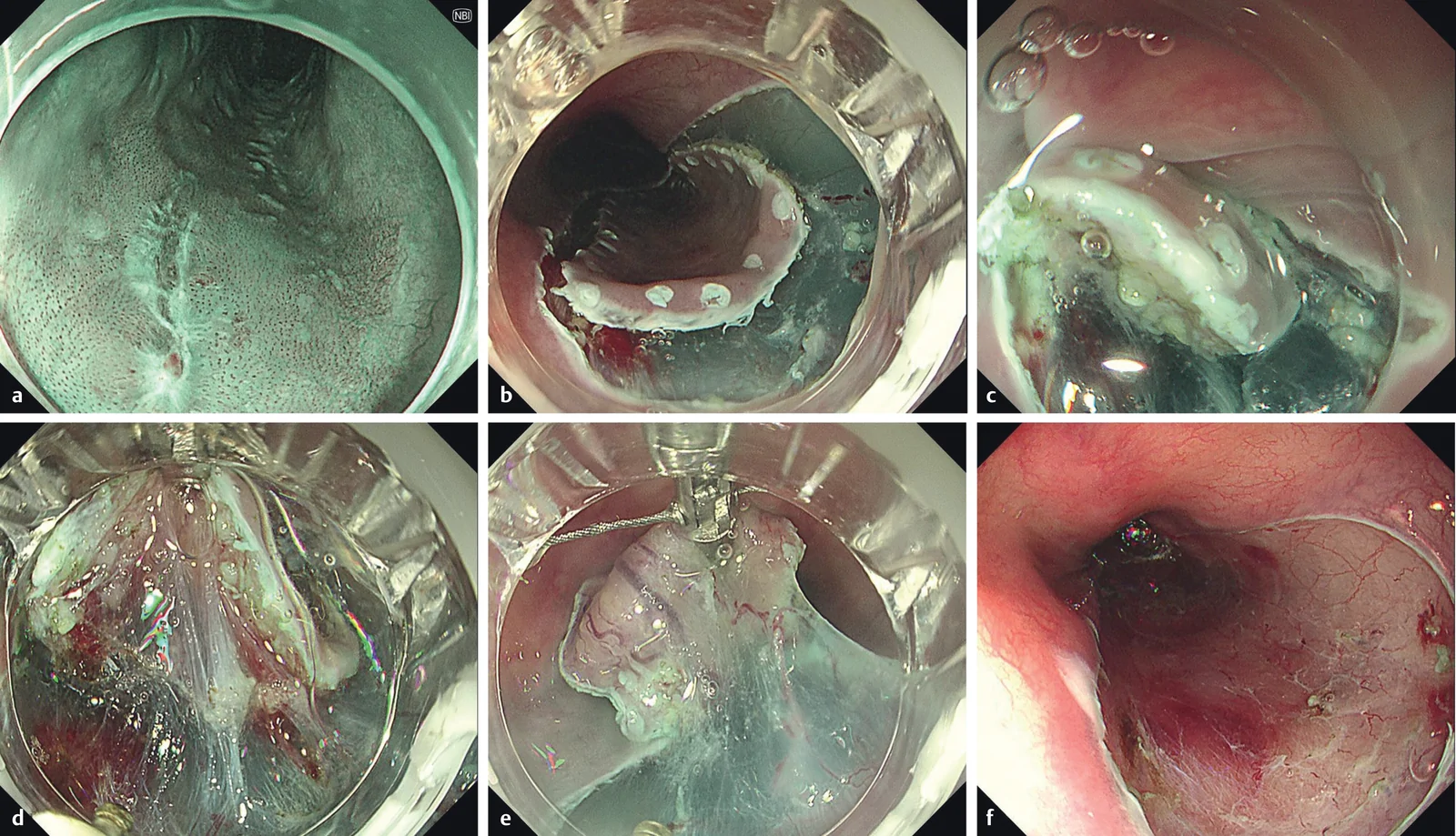
Endoscopic findings during EndoFaster-assisted ESD. ( **a** ) Narrow-band imaging (NBI) showing a 4.0×3.0 cm early esophageal cancer involving nearly half of the circumference on the left lateral wall of the lower esophagus. ( **b** ) Circumferential marking and incision performed with a hybrid knife. ( **c** ) Gravity-dependent fluid pooling during dissection, persistently obscuring the submucosal layer. ( **d** ) Robotic traction providing sustained upward pull, fully exposing the submucosal layer. ( **e** ) Blood vessels rendered clearly visible under dynamic multi-angle traction. ( **f** ) Post-resection ulcer bed after en bloc removal, with no evidence of perforation or active bleeding.

**Video 1** En bloc endoscopic submucosal dissection of an early esophageal cancer on the left lateral wall of the lower esophagus using a novel robotic traction system.


**Fig. 2:**
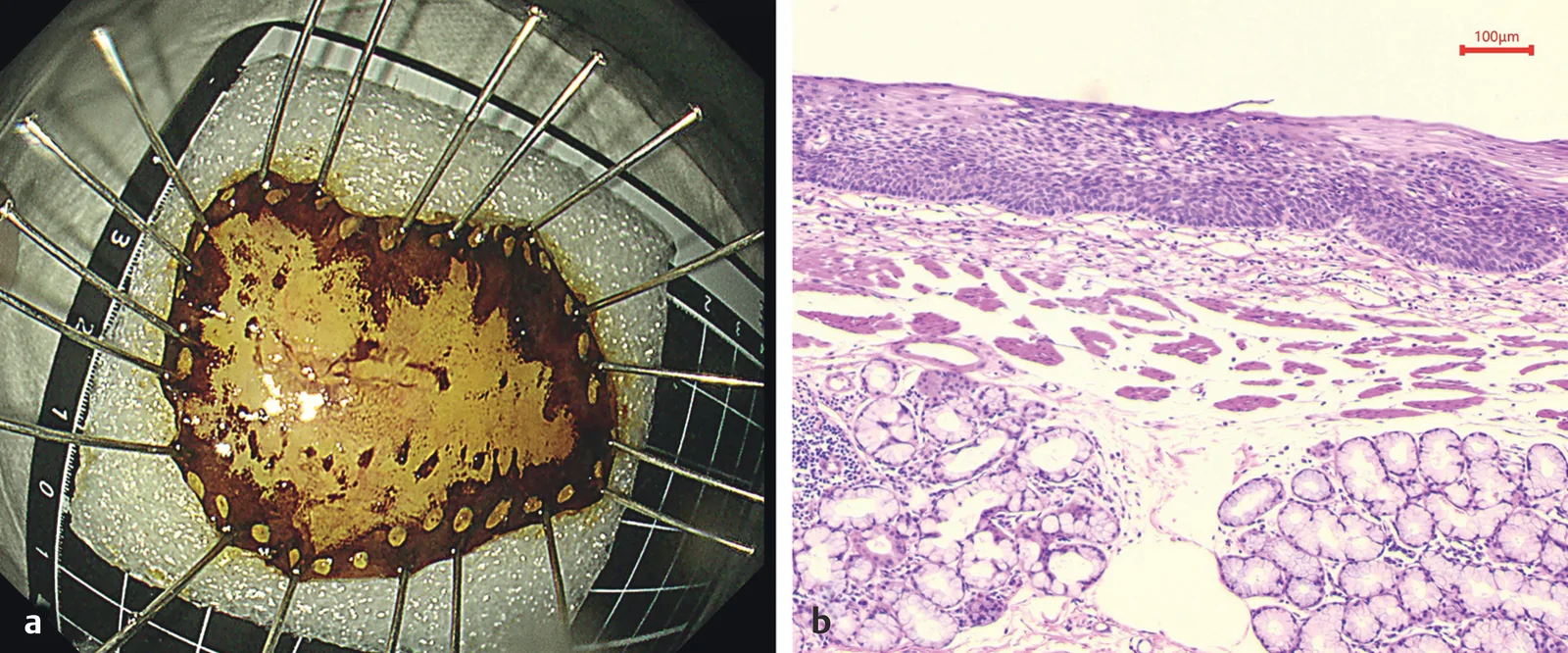
Resected specimen and histopathology. ( **a** ) The en bloc resected specimen pinned flat on a measuring grid. ( **b** ) HE (×100) confirmed squamous high-grade intraepithelial neoplasia with tumor-free lateral and vertical margins, supporting an R0 resection. HE, histopathological examination.

Although EndoFaster-assisted ESD has been reported in the stomach, rectum, duodenum, and esophagus, 1​ 2​ 3​ ​4​ the left lateral wall poses a particular challenge: gravity-dependent pooling obscures the submucosal plane, and conventional traction methods such as dental floss and the figure-of-eight clip 5​ pull only along the esophageal long axis and cannot lift the mucosa out of the fluid. EndoFaster’s multi-position, multi-angle traction overcomes this limitation, offering a strategy for safer ESD at gravity-dependent sites; further cases and follow-ups are needed to validate these advantages.

## Declaration of GenAI Use

The authors confirm that no generative artificial intelligence was used in the preparation of this manuscript.

Endoscopy_UCTN_Code_TTT_1AO_2AG_3AD
